# Phage N4 uses a SAR endolysin-holin system for host cell lysis

**DOI:** 10.1101/2025.11.12.688109

**Published:** 2025-11-12

**Authors:** Michael B. Awuah, Cody Martin, Jake S. Chamblee, Adam J. Tomaszewski, Teresa E. Sullivan, Qori Emilia, Steven Tran, Jason H. Snowden, Kaylyn A. Niemiec, Jiatong Zhu, Jolene Ramsey

**Affiliations:** 1 Department of Biology, Center for Phage Technology, Texas A&M University, College Station, Texas, USA; 2 Department of Biochemistry and Biophysics, Center for Phage Technology, Texas A&M University, College Station, Texas, USA; 3 Present address: University of Wisconsin, Madison, Wisconsin, USA; 4 Present address: Baylor College of Medicine, Houston, Texas, USA; 5 Present address: The Ohio State University, Columbus, Ohio, USA; 6 Present address: McGovern Medical School, University of Texas Health Houston, Houston, Texas, USA

**Keywords:** bacteriophage, membrane, lysis, N4, Schitoviridae, holin, endolysin, spanin, Escherichia coli

## Abstract

Bacteriophages (phages) cause active host cell lysis to terminate their infection and release progeny into the environment. Some phages delay their lysis, and thereby increase progeny yield, in a phenomenon called lysis inhibition (LIN). There are two dissimilar phages of *Escherichia coli* known to exhibit LIN: T4 and N4. Studies of phage T4 have demonstrated a multi-protein mechanism that stalls lysis and maintains the LIN state in response to superinfection in a high phage population density. However, the lysis proteins responsible for T4 lysis and LIN are not conserved with phage N4. In this study, we characterize the phage N4 proteins involved in lysis by molecular and genetic means. We define the functions of the minimal gene set required for lysis through heterologous expression and complementation. We also demonstrate that this complete lysis cassette is sufficient to induce LIN under high-density phage infection conditions. Furthermore, by sequence comparison with a selected mutant library that does not induce LIN, we have identified genomic regions both within and outside the lysis cassette involved in N4 LIN. We propose a model where N4 lysis proteins that are responsible for executing rapid N4 lysis can be regulated to induce LIN. Despite the lack of conservation with T4 components, our study suggests that direct modulation of lysis initiation may be common and provides a springboard for identifying other phages that regulate their phage production numbers in this way.

## Importance

Bacteriophages (phages) are viruses that only kill bacteria. To do so, they use lysis proteins targeted at the structural components of a bacterial cell. There are commonalities between the phages that infect bacteria with similar structures, but there are also interesting differences we can exploit to learn more about specific phages or their bacterial hosts. In this study, we characterize the lysis proteins a phage called N4 uses to kill *E. coli* bacteria. We also provide the basis for understanding how regulation of those proteins is linked to pathways that control phage yield by a mechanism that seems to differ from the one previously reported example in phage T4. Future studies on the relationship between lysis proteins and phage yield could provide handles to use this information for optimal phage production.

## Introduction

Characterization of host cell lysis in the golden age of phage biology revealed a multi-gene lysis (MGL) paradigm common among tailed phages (1). Phages gain evolutionary flexibility through regulatory interactions and mechanistic variations within their MGL systems (2–4). For example, coliphage N4, whose large, packaged virion RNA polymerase (vRNAP) is extensively and elegantly described from a biochemical perspective, modulates its yield by an order of magnitude higher via an unknown lysis regulation pathway (5). Being a tailed podophage with a 72 kb dsDNA genome, phage N4 is expected to encode a full suite of MGL proteins for progeny release.

A minimal set of MGL proteins includes the holin for initiating lysis and the endolysin for cell wall degradation (6). In diderm and mycolata hosts, specialized proteins such as spanins in Gram-negative bacteria are necessary for crossing the final host envelope barrier to progeny release (7–10). There are two major MGL systems in phages of Gram-negative bacteria: the holin-endolysin system and the pinholin-SAR endolysin system (1). In the canonical phage λ holin-endolysin-spanin system, pore-forming proteins known as holins accumulate harmlessly within the inner membrane. Upon reaching an allele-specific critical threshold concentration, holins depolarize the cell by making micron-sized holes (11, 12). Hole formation gives the muralytic endolysins access to their cell wall substrate in the periplasmic space. Degradation of the peptidoglycan meshwork liberates fusogenic proteins known as spanins to fuse the inner and outer membrane leading to cell envelope rupture (13). Similarly, in the phage 21 pinholin-SAR endolysin-spanin system, nanometer-scale pinholes depolarize the inner membrane (14). Pinholin action simultaneously releases signal-arrest-release (SAR) endolysins into the periplasm where they assume an active conformation (15). Due to the weakly hydrophobic nature of the SAR domain, SAR endolysins also stochastically leak from the inner membrane. When sufficient peptidoglycan is degraded, spanin action leads to explosive cell lysis. Many characterized tailed dsDNA phages infecting Gram-negative hosts encode an MGL system with these functions to release progeny across the cell envelope.

Despite the functional conservation across MGL systems, the MGL proteins exhibit high sequence variation. Holin proteins, for example, all encode a transmembrane domain, but require experimental characterization for accurate classification of their hole type. Therefore, detailed lysis studies are imperative to construct an accurate picture of the lysis landscape across phage and host cells. In phage N4, spanins were bioinformatically identified and an active SAR endolysin was reported in 2007, but holins are only predicted (16). Moreover, the N4 lysis inhibition (LIN) phenotype, an hours-long lysis delay resulting in higher phage yield, remains unsolved (17, 18). Phage T4 regulates its holin to drive LIN (19–21). The LIN system of phage T4 is also observed in T4-like phages (22). Without identified holins, phage N4 lacks a molecular framework upon which to study its LIN phenotype. Additionally, the past decade has witnessed a boom in reports of N4-like phages encoding the characteristic vRNAP (23–25). Although N4-like *Pseudomonas* phage AM.P2 may exhibit LIN, the N4-like coliphage G7C and *Achromobacter* phages JWDelta and JWAlpha do not exhibit LIN and most N4-like phage reports do not mention LIN at all (26–28). To better understand lysis and LIN within the N4-like phages, the phage N4 lysis system must be defined.

Here, we demonstrate that phage N4 encodes a complete and functional MGL system. Heterologous expression of individual predicted lysis proteins revealed their contribution to lysis. Expression of the full N4 MGL system leads to rapid lysis. The N4 master regulator pinholin and its lysis regulator protein usually co-occur in N4-like phages, suggesting commonalities in their lysis regulation pathways.

## Results

### The phage N4 predicted lysis genes cause rapid host lysis

**Figure 1.**
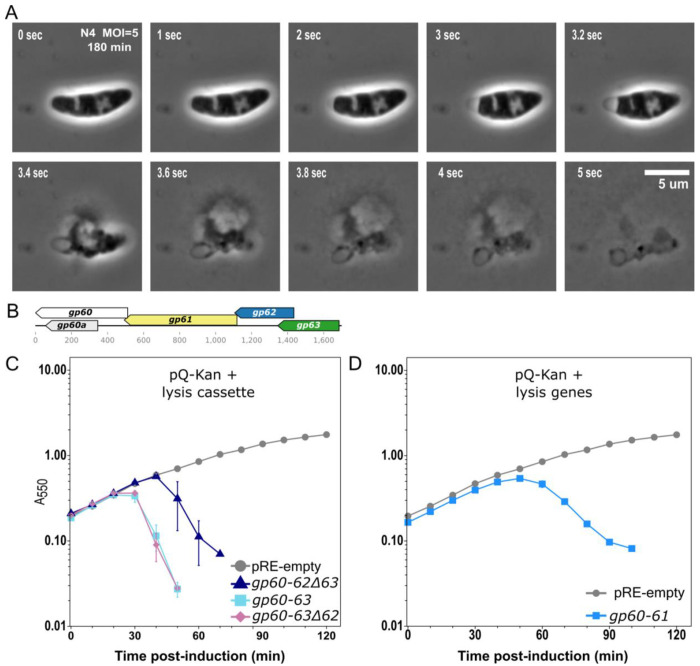
Phage N4 encodes a multi-gene lysis system capable of effecting rapid lysis. A) Representative time lapse microscopy of MG1655 180 minutes after infection with N4 WT at an MOI=5. Infected cells were imaged at 100x magnification. A complete time lapse video is Movie S1. B) Genetic organization of the phage N4 lysis genes including the i-spanin gp60, the embedded o-spanin gp60a, the endolysin gp61, the lysis regulator gp62, and the pinholin gp63. C) Lysis kinetics of MG1655 cultures expressing N4 lysis cassette genes from the pRE plasmid under the λ Q-dependent pR’ promoter. Host cultures harboring derivatives of pRE carrying N4 lysis genes and the pQ-Kan plasmid (λ Q under lacPO1 control) were induced with 1 mM IPTG at T=0 min. Lysis was monitored by optical density for pRE-empty (circle), gp60-62Δ63 (triangle), gp60-63 (square), or gp60-63Δ62 (diamond). D) Lysis activity with N4 endolysin and spanins alone was monitored for pRE-empty (circles) or gp60-61 which includes embedded gp60a (square). Lysis curves represent at least three biological replicates and data are presented as the mean ± standard error of the mean.

Although the wildtype (WT) isolate of N4 displays an asynchronous lysis in bulk culture starting ~3 hours after infection, a rapid lyser mutant completes synchronous lysis within an hour suggesting a functional MGL system (18, 29). We observed WT phage N4-infected host cells at times post-infection when population lysis was ongoing (Fig. 1A). Many cells were elongated and roughly rod shaped, suggesting that the endolysin had not been released. Additionally, when individual cells lysed, they did so rapidly and completely (Movies S1 & S2). Before lysis, each rod-shaped cell contracted from the poles, characteristic of SAR endolysin activity degrading the peptidoglycan evenly around the cell body after release from the inner membrane (1). This lysis pattern suggests that individual N4-infected cells lyse with the standard pattern expected for complete MGL systems, what we call rapid lysis.

Bioinformatic analysis of N4 and N4-like phages reveal clustered late genes encoding proteins with the classic characteristics of spanins and holins flanking the endolysin (Fig. 1B)(7). To demonstrate that the phage N4 WT genome encoded the genes necessary and sufficient to effect rapid lysis, we cloned the coding region encompassing gp60 and gp60a (putative embedded spanins), gp61 (SAR endolysin), and gp62-gp63 (putative holin/antiholin) into an inducible plasmid. Upon induction, we observed culture lysis between 20 and 50 minutes (Fig. 1C). These results demonstrate that N4 encodes a complete MGL pathway. The lysis pattern is unchanged when gp62 is deleted, suggesting that gp62 plays a nonessential role in lysis. Surprisingly, lysis onset and completion are only 20 minutes delayed when gp63 is deleted. These data suggest that gp63 controls the initiation of lysis timing. They also demonstrate that either gp62 or gp63 are sufficient to induce rapid lysis. In their absence, the leaky SAR endolysin gp61 paired with the gp60 and gp60a spanins resulted in slow culture lysis from 50-90 minutes post-induction (Fig. 1D). To further delineate the molecular roles of each gene in the lysis cassette, we proceeded to characterize their molecular properties.

### N4 encodes a SAR endolysin and spanins

**Figure 2.**
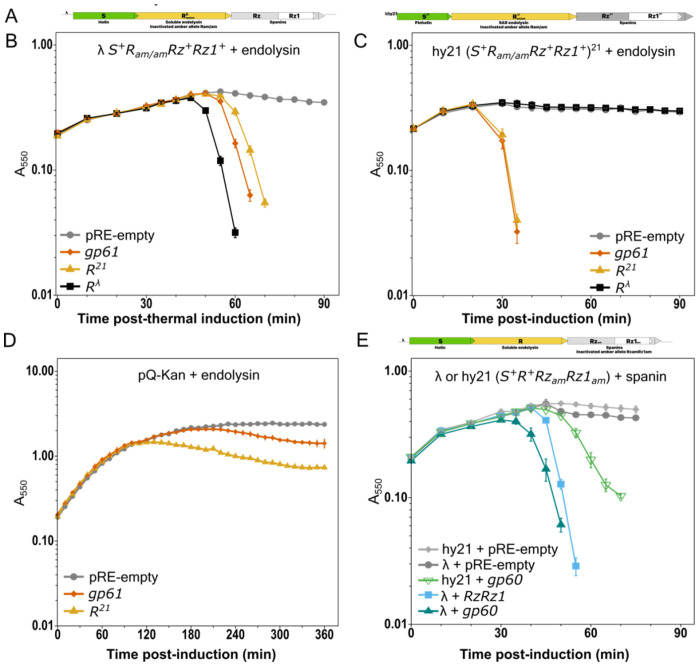
N4 endolysin and spanin function in lysis. **A)** Lysis gene schematics for the lysogen with an amber allele in the λ soluble endolysin R^λ^ or λ-phage 21 hybrid (hy21) SAR endolysin R^21^. **B)** Lysis gene activity in the λ lysogen with an amber allele in the soluble endolysin was monitored after thermal induction in the presence of pRE-empty (circle), N4 *gp61* (diamond), phage 21 endolysin *R*^*21*^ (triangle), or λ endolysin *R*^*λ*^ (square). **C)** As in B, lysis gene activity in the hy21 lysogen with an amber allele in the SAR endolysin after concurrent thermal and IPTG induction was monitored in the presence of pRE-empty (circle), N4 *gp61* (diamond), *R*^*21*^ endolysin (triangle), or *R*^*λ*^ endolysin (square). **D)** Lysis kinetics of MG1655 cultures with pRE -empty (circle), N4 *gp61* (diamond), or *R*^*21*^ (triangle) plasmids. **E)** As in B and C except the lysogens had amber alleles in the spanin genes. Lysis gene activity was monitored as above for pRE-empty (diamond, circle), λ spanins (*RzRz1*) (square), or N4 spanin *gp60* and *gp60a* (triangles). Lysis curves represent at least three biological replicates and data are presented as the mean ± standard error of the mean.

Prior studies reported the enzymatic activity of purified gp61 as a membrane-associated N-acetyl muramidase (16). Inspection of the N-terminal sequence revealed a weakly hydrophobic domain rich in glycine and alanine flanked by charged lysine residues, the characteristics of a SAR domain (Fig. S1). Indeed, when expressed in lysogens encoding either a canonical large hole or a pinholin but no SAR endolysin, gp61 complemented cell wall disruption activity (Fig. 2A and 2B). SAR endolysins are known to undergo independent stochastic release from the membrane and holin-dependent synchronous release (15). Gp61 expressed alone demonstrates low levels of cell growth inhibition, comparable to the levels observed with the phage 21 SAR endolysin, on a timescale that matches asynchronous exit from LIN (Fig. 2C). Both the leaky activity and functioning in conjunction with a pinholin confirms gp61as a SAR endolysin.

The genes downstream of gp61 were identified bioinformatically as the type members of a large spanin gene family (7, 30). The *gp60* inner membrane spanin (i-spanin) gene is predicted to encode an N-terminal transmembrane domain and 140 residues in the periplasm (Fig. S2). The *gp60a* outer membrane spanin (o-spanin) gene is predicted to encode a mature lipoylated protein of 56 residues (Fig. S2). Upon expression in spanin minus λ lysogens, N4 gp60+gp60a complemented outer membrane disruption (Fig. 2D). These experimental data confirm and expand upon earlier reports in the literature for the phage N4 SAR endolysin and spanins.

### N4 encodes a pinholin and lysis regulator

**Figure 3.**
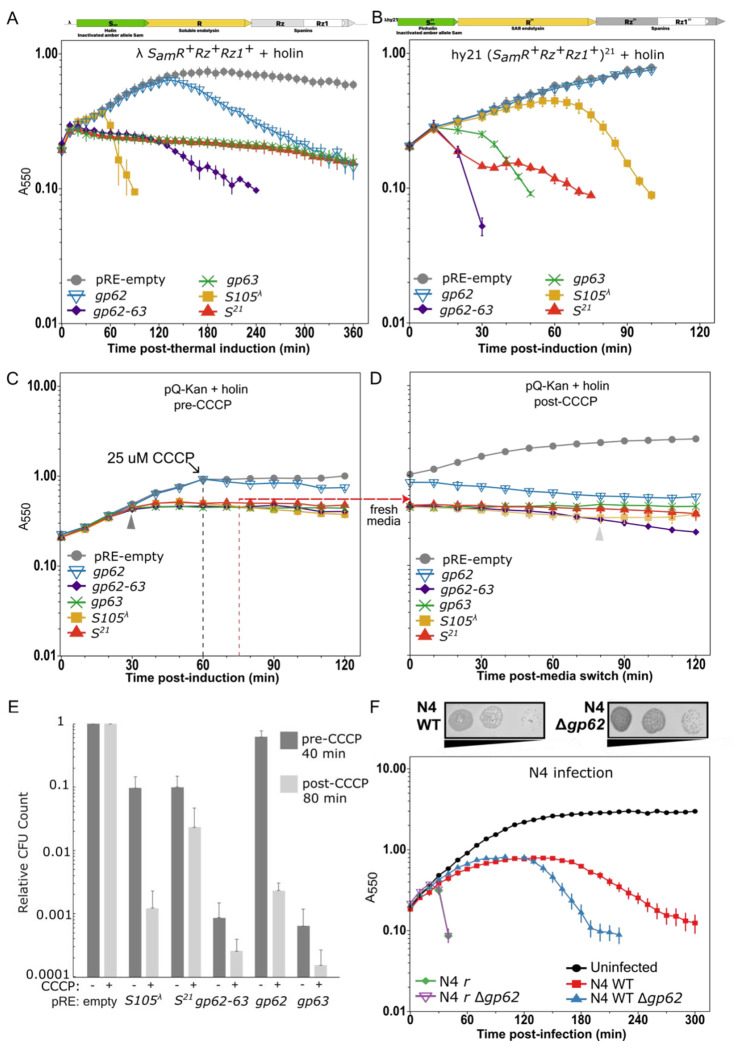
N4 encodes a holin and lysis regulator. Lysis was monitored in **A)** λ and **B)** hy21 holin amber lysogens as shown in schematic with simultaneous thermal and IPTG induction. **C) & D)** Lysis kinetics of MG1655 cultures induced with IPTG at T=0 minutes with or without 25 μM Carbonyl cyanide m-chlorophenylhydrazone (CCCP) treatment at 60 min (dashed grey line). Culture aliquots were removed, pelleted, and resuspended in fresh media without CCCP at 75 minutes (dashed red line). Strains in A-D harbor pRE-empty (circle), *gp62* (inverted triangle), *gp63* (x), *gp62*-*63* (upright triangle), *gp63* (x), *S105*^λ^ holin (square), or *S*^*21*^ pinholin (triangle). **E)** Relative colony-forming units (CFU) for each construct are normalized to the corresponding empty vector control at the respective time point, either 40- or 80-minutes post-induction from the experiment in C & D as marked by arrowheads. **F)** Dilution series of N4 plaques for the WT parent and *gp62* deletions in WT and the *r* rapid lyser on host lawns. An N4 infection at MOI=5 was monitored for WT (square), the *r* rapid lyser (diamond), and their *Δ62* derivatives (upright and inverted triangles, respectively). Lysis curves and viability counts represent at least three biological replicates and data are presented as the mean ± standard error of the mean.

Based on the canonical MGL model, N4 lysis is anticipated to initiate via a master regulator holin. Other than having nondescript transmembrane domains, holins lack conserved domains, necessitating experimental validation to definitely assign their function. The two transmembrane domain-containing proteins encoded upstream of the SAR endolysin are candidates. The *gp63* gene encodes a protein of 110 residues with an alternate start site at Met17. The full-length gp63 is predicted to be C-terminally anchored in the inner membrane (Fig. S3). Despite transmembrane domain prediction tools reporting multiple possible orientations, following the positive-inside rule we predict the longer N-terminal alpha-helical domain of gp63 remains in the cytoplasm. The *gp62* gene encodes a 109-amino acid protein with two transmembrane domains and both termini located in the cytoplasmic compartment (Fig. S3) (31, 32). Two alternative start sites with recognizable upstream Shine-Dalgarno sequences for *gp62* are reminiscent of the antiholin dual start motif regulatory module found in the λ S107/S105 gene. Though both N4 *gp62* and *gp63* genes encode alternative start sites, we selected for analysis the most conserved start sites with strong upstream ribosome binding sites. To investigate the activities of both gp62 and gp63, we expressed them individually and together in lysogens encoding either a soluble endolysin or a SAR endolysin (Fig. 3A and 3B). Gp63 demonstrated complementation of S^21^ pinholin activity only, suggesting it forms pinholes. Gp62 did not complement holin activity within the first two hours after induction when other characterized holins usually trigger hole formation. However, later gp62 alone did result in a gradual lysis of the bulk population. When gp62 and gp63 were expressed together in the soluble endolysin background, the cells ceased growth in under 30 min as seen for gp63 alone, and then asynchronous lysis began at 120 min as seen for gp62 alone, suggesting gp63 and gp62 both exert independent effects on the cell (Fig. 3A). In contrast, the gp62+gp63 coexpression in the SAR endolysin background resulted in an even more rapid decline in OD and CFU than either gene expressed alone, suggesting a synergistic effect (Fig. 3B). Due to this atypical behavior, we tested irreversible hole formation by artificially depolarizing the membrane with CCCP to trigger early hole formation (Fig. 3C and 3D). Gp63 behaved like the well-characterized λ holin S^λ^ and phage 21 pinholin S^21^. Although cells expressing gp62 alone demonstrated OD increase on par with control cells, they did not recover after CCCP treatment but instead had concomitant CFU decrease, suggesting irreversible hole formation (Fig. 3E). The cells overexpressing gp62, gp63, and both were unchanged in their rod morphology, similar to vector alone, S105, and S^21^ cells (data not shown). To further explore the role of gp62 in lysis during phage infection, we deleted this gene in the phage N4 genome and compared its lysis profile in a WT and rapid lysis mutant background. The *Δgp62* phage plaques were identical to the parent phage, however, asynchronous bulk culture lysis initiated an hour earlier than WT with a steeper slope (Fig. 3F). When expressing excess gp62 from a plasmid in both WT and r, we observed no change in lysis timing or plaque morphology (data not shown). Overall, these data demonstrate that gp63 is a pinholin. Since gp62 exhibits characteristics of both a holin and a holin regulator, we are designating it a lysis regulator.

### Mutations in the pinholin and SAR endolysin lead to rapid lysis

Given the presence of a fully functional MGL system, we wondered what lysis regulators prevented rapid lysis during phage N4 infections. Shortly after its initial discovery in the early 1960’s, nitrous acid-induced chemical mutagenesis of N4 led to the identification of the N4 *r* (for rapid lysis) phenotype (29). The original phage N4 rapid lyser exhibits synchronized culture lysis within an hour, contrasting with the asynchronous WT N4 lysis starting a few hours after infection. We acquired an N4 stock passaged from the early *r* strain. After plaque purification, we sent two lines from that stock for whole genome sequencing (summarized in Table S1). Mutations were observed scattered across the *r* genome, including silent mutations in the vRNAP gene gp50, changes in the length of a homopolymeric pyrimidine tract within the direct terminal repeat (DTR), multiple changes in noncoding regions, and missense mutations in middle and late genes. Since the separation between the original WT and *r* lines may have been exaggerated over time due to lab passaging, we could not assign a specific allele to the *r* phenotype with these data.

**Figure 4.**
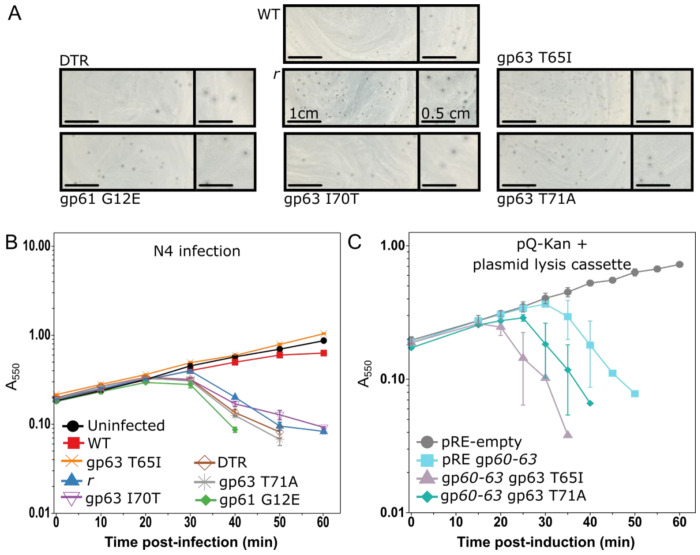
Lysis kinetics of N4 rapid lysers. **A)** N4 plaques for each rapid lyser class compared to the WT parent on host lawns. Right square image (0.5 cm scale bar) is magnified from left rectangle (1 cm scale bar). **B)** Lysis curve monitoring N4 MOI=5 infections for WT parent (square) and representatives of N4 rapid lyser classes: an original rapid lysis *r* derivative (upright triangle), DTR (open diamond), gp63 pinholin mutants (x, asterisk, and inverted open triangle), and gp61 SAR endolysin G12E (filled diamond). **C)** Lysis curve after 1 mM IPTG induction monitoring culture density for pRE-empty vector (circle), gp*60-63* (square), gp*60-63* gp63 T65I (triangle), or gp*60-63* gp63 T71A (diamond).

To build a comprehensive picture of the genetic changes that can lead to rapid lysis, we performed a selection for rapid lysing phage variants. Starting with the parent N4 WT, we collected infection supernatant before one hour of infection. Those supernatants yielded mixed plaque phenotypes matching both the smaller WT character and the larger *r* phenotype with fuzzy halos. Over 60 *r* phages were plaque-purified (representatives in Fig. 4A). Bona fide rapid lysers were subjected to targeted sequencing in the lysis cassette. Isolates with no changes in the lysis cassette were whole genome sequenced (summarized in Table 1). Representatives shown in Fig. 4B display rapid lysis like the original *r* line. We grouped the mutations into four Classes: lysis cassette mutants, mostly silent mutants in the vRNAP gp50 gene, DTR mutants, and miscellaneous changes. Characterization of the vRNAP and DTR Classes will be reported separately (manuscript in preparation). The miscellaneous Class included changes found throughout the genome in both coding and noncoding regions, some co-occurring with the other classes, that were not isolated more than once. One of the miscellaneous Class missense lines was Q98R in gp53, a late gene whose protein product is predicted to contain transmembrane domains. We built the gp53 Q98R change into a clean N4 WT background, but it did not replicate the rapid lysis phenotype (data not shown).

Here, we focus further on the mutations in the lysis proteins gp63 and gp61. The two changes in gp61 introduced a single negatively charged residue into the SAR domain, likely preventing its anchoring in the inner membrane and leading to rapid lysis. The six changes identified in gp63 are clustered in a highly conserved region near the transmembrane domain. Five of the six mutations resulted in changes to or from serines and threonines. To verify that these changes are involved in rapid lysis, we reconstructed two gp63 alleles in the plasmid lysis system. Although the *gp63* T65I allele demonstrated variable lysis phenotypes in the rapid lyser isolates, both T65I and T71A *gp63* alleles demonstrated rapid lysis faster than the parent lysis cassette (Fig. 4C). Due to the clustering of the gp63 changes, we designated this region the Receiver domain and postulate it serves a regulatory function, possibly mediated by gp62 although our screening of over 60 individual plaques did not isolate a single gp62 mutant. Consistent the SAR endolysin and pinholin genes being important for regulating lysis timing, we found multiple alleles for each resulted in rapid lysis.

### Conservation of the N4 lysis proteins

**Figure 5.**
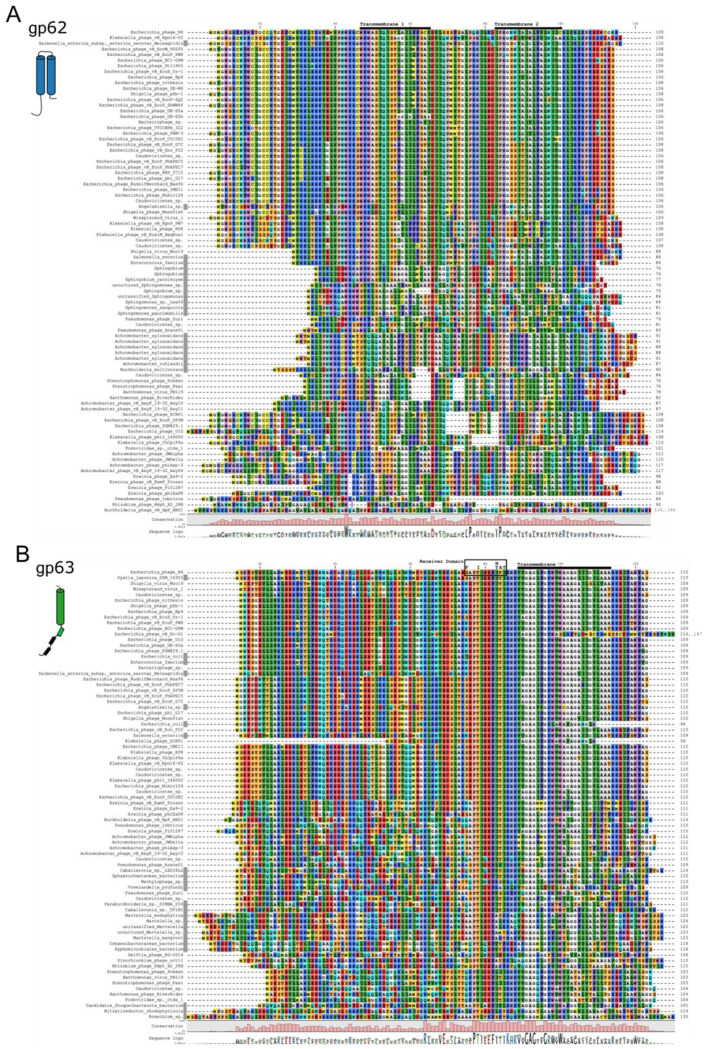
Protein alignment shows conservation in N4 pinholin and lysis regulator. Multiple sequence alignment of proteins with significant sequence similarity to the N4 lysis regulator gp62 (A) and pinholin gp63 (B) by BLASTp and BLASTx. Sequences in Rasmol colors are labeled by their source organism with bacteria marked by a gray box after the organism name. Predicted transmembrane regions are marked by a black bar across the top and mutations identified in gp63 rapid lysers are shown within the boxed Receiver domain.

Among all the 144 high-quality cultured N4-like virus contigs identified with at least seven core genes (25), the lysis proteins are not identified as highly conserved. Using BLASTp and BLASTx we searched for bacterial or phage proteins with sequence similarity to gp63 or gp62. The hits were distributed across bacterial assemblies and N4-like phages, plus one plasmid contig. Many of the bacterial contigs, like the plasmid, were approximately the size of a complete N4 genome, suggesting a function lysis cassette would be present. For phages with BLAST hits to only gp62 or gp63, manual genome inspection usually revealed a misannotated or unannotated pair protein that we added to our list. Gp62 and gp63 co-occurred in 60/65 N4-like genomes with hits (Table S2). After deduplication to remove identical sequences, protein alignments for gp62-like proteins showed conservation of the two transmembrane domains with a few residues conserved in nearly 100% of the 86 aligned proteins (Fig. 5A). The deduplicated alignment with 80 gp63-like proteins showed a variable N-terminal cytoplasmic tail followed by highly conserved transmembrane and Receiver domains (Fig. 5B). Their co-occurrence and conserved transmembrane and Receiver regions are consistent with a regulatory role for gp62 in gp63-mediated lysis events.

## Discussion

**Figure 6.**
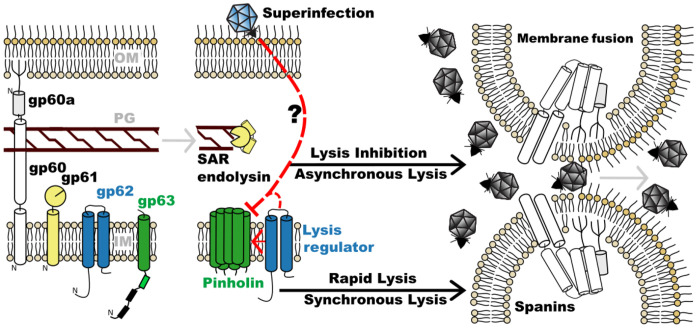
N4 lysis model. Phage N4 lysis proteins, the spanins (gp60 and gp60a), SAR endolysin (gp61), lysis regulator (gp62), and pinholin (gp63) can effectuate release of the SAR endolysin to cause the rapid and synchronous lysis phenotype seen in *r* phage. In the presence of superinfection, leaky SAR endolysin activity possibly mediated by the gp62 lysis regulator leads to asynchronous lysis at later times to release phage from cells in the LIN state.

Here, we demonstrate that phage N4 has a fully functional MGL lysis system composed of a pinholin, SAR endolysin, bimolecular spanins, and a lysis regulator. Though WT phage N4 exhibits LIN with a long asynchronous lysis, expression of the N4 MGL system from a plasmid effects rapid, synchronous culture lysis. By selecting for phage exhibiting rapid lysis, we isolated spontaneous rapid variant alleles including in the phage pinholin and endolysin genes. Therefore, our results suggest that N4 pinholin regulation controls phage entry into LIN. To explain how phage N4 exits the LIN state, we propose a model where phage release is mediated by either lethal lysis regulator activity or stochastic release of the SAR endolysin (Fig. 6).

Due to the weakly hydrophobic SAR anchor, slow rates of stochastic SAR endolysin release can lead to gradual population lysis. As in phages P1, 21, and Mu, the N4 SAR endolysin inner membrane anchor sequesters its catalytic residues until pinholes release them from the membrane (15, 33). The phage Mu SAR endolysin shows minimal release at near-native expression levels, but overexpression or the presence of its regulator causes rapid release and cell lysis (34). Upon overexpression, N4 gp61 results in complete cell lysis and prior unpublished studies on gp63 as an activator of gp61 are consistent with these findings (16, 35). The multiple alleles of gp63 with altered lysis timing support its assignment as a holin (Fig. 4B). Additionally, rapid lysis could be induced late into the LIN state, suggesting that N4 regulatory factors are responsible for inhibiting lysis (18). Observations of individual cells during asynchronous population lysis revealed the characteristic rounding cell shape associated with SAR endolysin degradation of the cell wall prior to cell bursting (Fig. 1 and Movies S1 and S2). These data support a role for the leaky SAR endolysin in both lysis and exit from LIN.

Alternatively, lethal activity from accumulated gp62 may induce SAR endolysin release. We classified the gp62 protein as a lysis regulator because its associated phenotypes are consistent with multiple activities. First, the N4 *Δgp62* phage resulted in earlier lysis, consistent with antiholin behavior (Fig. 3F). Second, Gp62 displayed holin-like behavior when expressed from a plasmid. Gp62 promoted rapid lysis from the plasmid system (Fig. 1C), is independently toxic to the cell (Fig. 3C-E), and released soluble λ endolysin, a globular enzyme of 17.8 kDa, across the IM in an asynchronous fashion with delayed onset (Fig. 3A). Although gp62 alone did not release the phage 21 SAR endolysin after two hours, its other phenotypes suggest the possibility of later activity. These data suggest gp62 may be an alternative holin or cause general membrane perturbation. Third, Gp62 appears to promote gp63 pinholin activity as observed when gp62+gp63 and the SAR endolysin result in rapid synchronous lysis (Fig. 3B). gp62 could promote gp63 activity as a separate holin or through nonspecific membrane permeabilization since protonophores are known holin triggers and some holin-like proteins promote nonlytic secretion (36). Altogether, these data suggest gp62 is a nonessential lysis regulator that influences exit from LIN.

There is precedent for nonessential lysis regulators influencing lysis via independent toxic activity. Phage P1 encodes multiple holin-like proteins that provide flexibility in varied environments (37–40). Toxicity after the genetically programmed holin trigger time has also been observed with the antiholins of class I holins and class III pinholins (41). The λ S107 antiholin is produced by use of an alternative start site with only two amino acids unique at the N-terminus. When expressed together at the native 2:1 ratio, S107 delays S105 lysis time (32). When expressed alone or after addition of energy poisons, the S107 antiholin is toxic to the cells, albeit later. The toxicity gp62 manifests more than an hour after an uninhibited gp63 pinholin triggers lethal hole formation coincides with the time when N4 WT exit from LIN via asynchronous lysis begins (Fig. 3B). Although our bioinformatics showed that most phages encode gp62 and gp63 as a pair, this does not preclude an independent gp62 activity in LIN exit.

The N4 lysis proteins differ from the T4 lysis proteins that lead to LIN. In phage T4, LIN activates when superinfecting phage DNA trapped in the periplasm binds to and stabilizes an antiholin-holin complex (20, 21, 42, 43). The phage T4 holin T, a class III single transmembrane domain holin, has a globular domain that interacts with soluble rI antiholin in the periplasm (19, 21). A set of periplasmic and membrane-bound superinfection exclusion proteins intercept secondary T4 phage infections (44). The T interaction with rI and intercepted superinfecting phage DNA in the periplasm leads to LIN. Although both the superinfection exclusion and lysis proteins triggering LIN are widespread among T4-like phages, the N4 lysis proteins are not similar to phage T4. Although a superinfection exclusion system is not characterized for phage N4, prior reports are consistent with superinfection leading to LIN (29). However, gp63 is a class III pinholin with an N-in C-out membrane orientation, placing its Receiver domain in the cytoplasm. The gp62 lysis regulator also orients its soluble termini toward the cytoplasm, strongly suggesting a completely different mechanism of superinfection detection and LIN in phage N4. Therefore, we hypothesize that in N4 a superinfecting phage signal directly inhibits the gp63 pinholin via an interaction at its cytoplasmic Receiver domain during entry. Identification of the superinfection exclusion system in N4 will greatly advance our understanding of what factors influence entry into LIN.

We demonstrated that N4 lysis proteins cause rapid lysis (Fig. 6). With the primary lysis proteins identified, the other rapid lysis mutant classes reported here will be mined for these inhibitory factors. Elucidating the relationship between N4 lysis and will reveal the mechanistic underpinnings connecting lysis timing and phage yield in a phage with an average burst size of ~3000 (45). Further comparisons among diverse N4-like and other phages could reveal common and unique principles that govern phage yield.

## Materials and methods

### Bacterial strains and growth conditions

All *Escherichia coli* MG1655 derivatives, plasmids, and phages used in this study are listed in (Table 2). Cultures were grown in lysogeny broth (LB) medium, composed of 10 g/L tryptone, 5 g/L yeast extract, and 10 g/L NaCl (no magnesium supplementation) with aeration at 37°C, except lysogens which were maintained at 30°C until thermal induction. Solid medium for plates was prepared by adding 1.5% (w/v) agar. For phage plaque assays, a soft agar overlay consisting of LB with 0.75% (w/v) agar was used. Strains harboring plasmids were maintained under appropriate antibiotic selection: ampicillin at 100 μg/mL, kanamycin at 40 μg/mL, or chloramphenicol at 10 μg /mL. Glucose at 0.4% (w/v) was added to repress leaky lysis protein expression in their respective cultures.

For infections and inductions, a saturated host culture was subcultured at a 1: 200 dilution in 25 mL of LB and grown to a mid-log absorbance at 550 nm (A_550_) of approximately 0.2, then induced or infected. For phage infections, mid-log cultures were incubated on ice during a 10-minute adsorption. Unless otherwise stated, infections were performed at a multiplicity of infection (MOI) of 5. For thermal induction, lysogens were grown with aeration at 30°C, shifted to 42°C for 15 minutes to induce the prophage, then transferred to 37°C. For chemical induction of plasmid-borne phage genes, 1 mM isopropyl-β-D-thiogalactopyranoside (IPTG) was added. λ lysogens were only thermally induced as the Q antiterminator produced from the phage genome will antiterminate the pR’ transcripts from the pRE vector. Hy21 lysogens encode the phage 21 Q, therefore they were paired with the pQ plasmid carrying λ Q and induced both thermally and with IPTG to antiterminate transcripts of the pRE vector. Lysis experiments were performed at least three times and the data are presented as the mean ± standard error of mean.

### Construction of plasmids and phages

The predicted lysis cassette of phage N4 was PCR amplified and cloned into restriction sites BamHI and KpnI in the pRE vector by ligation. Clones were verified by Sanger sequencing with Eton Bioscience Inc (San Diego, CA) or Plasmidsaurus Inc. (Louisville, KY). Individual N4 genes were cloned in the same manner using oligos listed in Table 3 to generate plasmids listed in Table 2. To generate the gp63 rapid lyser alleles in the pRE plasmid lysis cassette, the full lysis cassette plasmids were amplified with primers containing the base changes to generate T65I and T71A by overlap PCR. The λ R clone in pRE had an mCherry fusion removed via omission PCR followed by ligation.

The N4 gp62 deletion was constructed by WT infection of host cells containing the pRE gp*60-63Δgp62* plasmid at an MOI=5 or 0.1 (both yielded positive recombinants). Cross-lysate was collected and sterilized after an overnight incubation. The parent phages in cross-lysates were counterselected against on lawns with pBA560 encoding eLbuCas13a and a spacer targeting gp62 (constructed from oligos 5’- aaacagaactggataagacatccacacaagagaagA- 3’ and 5’- agcatcttctcttgtgtggatgtcttatccagttct -3’) with 0.6 uM anhydrous tetracycline in three rounds of plaque purification, according to the method of Adler *et al.*(46). The pBA560 plasmid was a gift from Jennifer Doudna (Addgene plasmid # 186236; http://n2t.net/addgene:186236; RRID:Addgene_186236). Whole genome sequences were verified using Illumina services at Microbial Genome Sequencing service, now SeqCenter LLC (Pittsburgh, PA), or Plasmidsaurus Inc. (Louisville, KY) Oxford Nanopore Technology long-read sequencing services.

### Plaque morphology and imaging

To visualize plaque morphology, 100 μL of phage dilution was mixed with 100 μL of a saturated overnight host culture. The mixture was incubated on ice for 10 minutes to facilitate phage adsorption. Subsequently, the phage-bacteria mixture was added to 4 mL of molten soft agar and immediately poured onto an LB agar base plate. Plates were incubated overnight at 37°C until plaques developed. Representative plaques were then photographed using an iPhone 14 Pro Max camera set to 2.5X Portrait Mode.

### Microscopy of lysing cells

Phage-infected cells were placed directly from a shaking culture onto a glass slide under a coverslip. Time series observations were acquired with an Axiocam 702 mono camera mounted on a Zeiss Axio Observer 7 inverted microscope using the alpha plan-apochromat 100×/1.46 oil (UV) Ph3 oil M27 objective. Exported videos were processed using the Carl Zeiss Zen Blue 2.3 imaging software.

### Rapid lysis phenotype selection and genotyping

Spontaneous mutations leading to rapid lysis were selected in two ways. Initially, spontaneous larger plaques with halos that resemble *r* plaques were plaque-purified directly from plates of WT phage. This method was low frequency and not predictable, but rapid lysers were obtained by this method. Second, infected culture supernatant was collected at 20-, 30-, or 60-minutes post-infection to enrich for early alleles. This was repeated up to three times followed by plaque purification.

Targeted sequencing was performed by Sanger sequencing of lysis cassette PCR amplicons using the oligos listed in Table 3. For rapid lysers with no mutations in the lysis cassette, phage genomic DNA was extracted from PEG-precipitated virions and sequenced by Illumina as described previously (47). Assembly and annotation were completed in the Center for Phage Technology Galaxy bioinformatics suite followed by consensus analysis in SnapGene v8.1 (48). Reads were mapped and analyzed for differences using Snippy at usegalaxy.eu (49).

### Holin-CCCP assays

Fresh overnight cultures were diluted, grown, and induced as described above. Sixty minutes post IPTG-induction, holins were triggered by the addition of Carbonyl cyanide m-chlorophenylhydrazone (CCCP) to a final concentration of 25 μM. Fifteen minutes after CCCP addition, 15 mL of the culture was pelleted, the supernatant was removed, and the cell pellet was resuspended in 15 mL of pre-warmed LB containing 1 mM IPTG. This culture was then aerated and monitored simultaneous with the initial flask.

To assess bacterial viability before and after CCCP treatment, samples were collected at two time points: 40 minutes post-induction (prior to CCCP addition) and 80 minutes after induction in cultures where CCCP had been removed. At each time point, 100 ul of culture was serially diluted in fresh LB. For each sample, 100 μl of a suitable dilution was plated in triplicate and incubated overnight at 37°C. Colonies were counted and expressed as relative CFU counts compared to the matched pRE-empty control collected at the same time point. CCCP experiments were performed in triplicate and the data are presented as the mean ± standard error of mean.

### Protein sequence analyses

Similar protein sequences were identified through BLASTx and BLASTp searches with the gp62 109 amino acid sequence and gp63 110 amino acid sequence against the non-redundant protein sequences database (retrieved May 4, 2025) and clustered using CD-HIT for 100% identity clusters (50–52). Phages without both gp62 and gp63 hits were manually inspected to find missing protein pairs and accessions or coordinates were added to Table S2. All gp62 hits were inspected for an extended N-terminal sequence missed in many annotations. The resulting sequences were aligned using CLC Main Workbench 25.0.1 Manual adjustments were made to an initial alignment performed with gap open cost 10, gap extension cost 1, and end gap cost free (very accurate alignment setting) settings.

Structure prediction used the AlphaFold3 Server (https://alphafoldserver.com/) and ChimeraX 1.9 (53, 54). Comprehensive topological and domain assignment was performed by integrating multiple prediction algorithms. Transmembrane helices were identified using TMHMM 2.0 while N-terminal signal peptides and their associated cleavage sites were predicted with SignalP 6.0 (55, 56). SignalP was run selecting “other” for organism on the slow model mode. Unless otherwise stated, all bioinformatic analyses used default parameters.

### Figure preparation

Data were graphed using Microsoft Excel or a custom R Shiny application developed with assistance from artificial intelligence (Claude Sonnet 3.7 by Anthropic & Grok 3 by xAI). The Shiny application was built using R version 4.5.0 with the following packages: shiny, tidyverse, ggpubr, gridExtra, scales, ggrepel, ggprism, svglite, and jsonlite. The code and instructions for its use are available at (https://github.com/mbaffour/N4-Lysis-paper-codes). Graphics for the figures were assembled in Inkscape 1.3.2 (https://inkscape.org/).

## Supplementary Material

Supplement 1

Supplement 2

3

## Figures and Tables

**Table 1.** Rapid lyser mutant classes. Summary of spontaneous genetic changes associated with rapid lysis in N4 after selection for early lysis in liquid and/or large *r* plaque phenotype. All nucleotide changes are given relative to the coding strand, which refers to the nontemplate strand of the open-reading frame for a gene, or the plus strand if not a coding region.

	Genomic Feature	Nucleotide change(s) by coding strand (+) or (−)	Coding change

**Class I:** Lysis genes	Gp61 SAR Endolysin	(−) GGG>GAG	G12E
		(−) GGA>GAA	G13E

	Gp63 Pinholin	(−) TCT>TTT	S62F
		(−) ACT>ATT	T65I
		(−) ATT>AAT	I70N
		(−) ATT>ACT	I70T
		(−) ACC>GCC	T71A
		(−) ATC>CTC	I72L

**Class II:** vRNAP	Gp50 vRNAP	(−) TCT>TCA	S781S
		(−) TTG>TTA	L786L
		(−) TTG>TTA	L808L
		(−) CTT>CTA	L828L
		(−) TTG>TTA	L841L
		(−) CTG>CTA	L878L
		(−) CAT>TAT	H882Y

**Class III:** Miscellaneous, co-occur with other classes, no repeat isolations	Gp1 Gp39	(+) ACT>AC_C_	T3T
		(+) **T**GG>CGG	W62R

**Class IV:** DTR changes	Noncoding cytosine run	Expansion or contraction of 9-14 Cs in DTR	-

**Table 2.** Phage, bacteria, and plasmids used in this study.

Name	Genotype and relevant features	Reference or source
**Phages**		
N4 WT parent	Virulent podophage of *E. coli* K-12	Gift from Ian Molineux, (57)
N4 *r*	N4 rapid lyser, originally isolated after nitroguanosine mutagenesis based on plaque size	Gift from Ian Molineux, (29)
N4 Δ*gp62*	215 bp deletion including gp62 residues T28-L99, derived from N4 WT	This study
N4 *r* Δ*gp62*	215 bp deletion including gp62 residues T28-L99, derived from N4 *r*	This study
		
**Bacteria**		
*E. coli*	MG1655 Δ*fhuA lacI^q^* Δ*lacY*	Lab stock
λ S_am7_ lysogen	MG1655 Δ*fhuA lacI^q^* Δ*lacY* λ *stf::cam cI_857_* *S_W56am_R*^+^*Rz*^+^*Rz1*^+^ *bor::kan*	Lab stock derived from (58)
Hy21 S^21^_am_ lysogen	MG1655 Δ*fhuA lacI^q^* Δ*lacY* λ *stf::cam cI_857_* hy21 (*S_am_R*^+^*Rz*^+^*Rz1*^+^)^21^ *bor::kan*	Lab stock derived from (59)
λ R_am/am_ lysogen	MG1655 Δ*fhuA lacI^q^* Δ*lacY* λ *stf::cam S*^+^*R_Q26am/W73am_ Rz*^+^*Rz1*^+^	Lab stock derived from (58)
Hy21 R_am/am_ lysogen	MG1655 Δ*fhuA lacI^q^* Δ*lacY* λ *stf::cam cI_857_* hy21 (*QS68*^+^*R_am/am_Rz*^+^*Rz1*^+^)^21^ *bor::kan* pQ-Cam	Lab stock derived from (59)
λ Rz_am_Rz_1am_ lysogen	MG1655 Δ*fhuA lacI^q^* Δ*lacY* λ *stf::cam cI_857_* hy21 (*S*^+^*R*^+^*Rz_Q100am_Rz1_W38am_*)^21^ *bor::kan*	Lab stock derived from (58)
Hy21 Rz_am_Rz1_am_ lysogen	MG1655 Δ*fhuA lacI^q^* Δ*lacY* λ *stf::cam cI_857_* (*S*^+^*R*^+^*Rz^am^ Rz1_am_*) *bor::kan*	Lab stock derived from (59)
		
**Plasmids**		
pQ	λ Q cloned under P_lac/ara-1_ promoter in a low copy number plasmid pZS-24	(60)
pQ-Cam	pQ plasmid encoding chloramphenicol acetyltransferase	(60)
pQ-Kan	pQ plasmid encoding aminoglycoside phosphotransferase from Tn5	(60)
pRE-empty	Medium copy plasmid containing Q-dependent λ late promoter pR’	(61)
pRE N4 gp60	N4 genes *60* and embedded *60a*	This study
pRE N4 gp61	N4 gene *61*	This study
pRE N4 gp62	N4 gene *62*	This study
pRE N4 gp63	N4 gene *63*	This study
pRE N4 gp62-63	N4 genes *62* & *63*	This study
pRE N4 gp60-61	N4 genes *60, 60a,* & *61*	This study
pRE N4 gp60-63	N4 genes *60*, *60a*, *61*, *62*, & *63* (lysis cassette 1678 bp)	This study
pRE N4 gp60-62Δ63	N4 genes *60*, *60a*, *61* & *62* (lysis cassette 1427 bp)	This study
pRE N4 gp60-63Δ62	N4 genes *60*, *60a*, *61*, & *63,* 215 bp deletion including gp62 residues T28-L99	This study
pRE N4 gp60-63 gp63 T65I	Derivative of pRE N4 gp60-63	This study
pRE N4 gp60-63 gp63 T71A	Derivative of pRE N4 gp60-63	This study
pRE R	R^λ^	This study
pRE R21	phage 21 R^21^	This study
pRE RzRz1	λ RzRz1 (96% & 98% identity to phage 21 RzRz1)	(62)
pRE S105	λ S105-sfGFP	(63)
pRE S21	phage 21 S^21^-sfGFP	Lab stock
pBA560-Cas13a-gp62	Encodes eLbuCas13a and spacer targeting gp62	Gift from Jennifer Doudna, Addgene plasmid # 186236; (46)

**Table 3.** Oligonucleotide sequences used in this study.

Primer	Sequence (5′–3′)
N4 gp60 KpnI Forward	CTAACCGTATTGGTACCGTCCCTCTGGAGAGGTGTAATG
N4 gp60 BamHI Reverse	CTGGCAATGGAACCATAGAATCAGTGTTGGATCCATAGTAATTCC
N4 gp61 KpnI Forward	TATTTGCCGACTGGTACCTTTGAAACCAAAGGAGGATTG
N4 gp61 BamHI Reverse	AGTACTCCTTCTTATCTGGATCCCATAGTGCTTGAACTTTCTTTTC
N4 gp62 KpnI Forward	GAAGAGTTCGGTACCATCAAACACAAAGTTGTTGGTGC
N4 gp62 BamHI Reverse	CAGCAATGATTGCTGCAATGGATCCCCCAACACCAC
N4 gp63 KpnI Forward	CTATTTGACGGGTACCTAAACACACTCTCTGTTCATTAGGACTAACAGAGC
N4 gp63 BamHI Reverse	CAACGTTTCCAATTAGGAACCGGATCCACATGTTTGGTTAAC
N4 gp63 T65I Forward	CTGAGTCTGCCCCTATTATTGAAGAGTTCATTACCATC
N4 gp63 T65I Reverse	GATGGTAATGAACTCTTCAATAATAGGGGCAGACTCAG
N4 gp63 T71A Forward	CCTACTATTGAAGAGTTCATTGCCATCAAACACAAAGTT G
N4 gp63 T71A Reverse	CAACTTTGTGTTTGATGGCAATGAACTCTTCAATAGTAGG
R-mCherry deletion Forward	TACATCAATCTCTCTGACCGTTCC
R-mCherry deletion Reverse	TAATAGTAGGGATCCGTCGACC
pRE N4 gp60-61 Forward	GAAACCAAAGGAGGATTGGTGTGG
pRE N4 gp60-61 Reverse	GAAAAAGCCGGGTATCCCCG
pRE N4 gp60-63 Delta 62 Forward	CCAAAGGAGGATTGGTGTGGCAATAA
pRE N4 gp60-63 Delta 62 Reverse	TTAACTTGCGAACCATGCAAAAATCTCC
pRE sequencing Forward	TTTTACACATGACCTTCGTGA
pRE sequencing Reverse	AGGCAAATTCTGTTTTATCAGA
N4 gp60 seq rev	CTTGCAAGGAAAGTTCATTC
N4 gp61 seq for	CCAGTTCTTTCTCTGCATTAC
