# Supplementary material for: Phage N4 uses a SAR endolysin-holin system for host cell lysis
Source: bioRxiv. 2025 Nov 12:2025.11.12.688109. Preprint. [Version 1] doi: 10.1101/2025.11.12.688109 (PMC12642591; doi:10.1101/2025.11.12.688109)
Supplement: 3 [file NIHPP2025.11.12.688109v1-supplement-3.pdf]

bioRxiv preprint doi: https://doi.org/10.1101/2025.11.12.688109; this version posted November 12, 2025. The copyright holder for this preprint (which was not certified by peer review) is the author/funder, who has granted bioRxiv a license to display the preprint in perpetuity. It is made available under aCC-BY 4.0 International license.

Supplementary Data

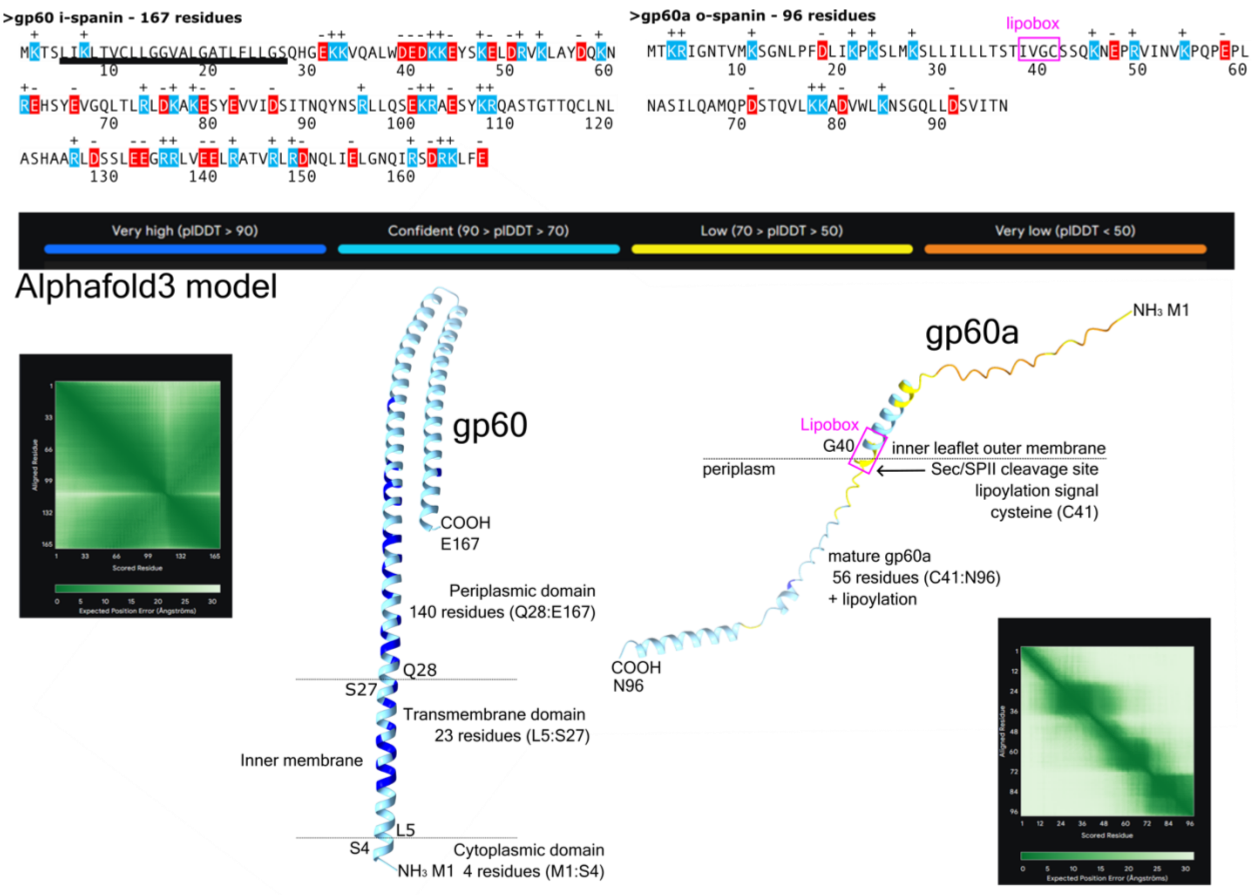

Figure S1. Predicted three-dimensional structure and topological organization of N4 SAR endolysin. The full primary sequence of gp61 with charged residues marked, catalytic triad residues boxed in neon green, and the SAR domain underlined. A tertiary fold model was predicted using the AlphaFold3 Server (top model shown), oriented relative to the respective membrane, and annotated with lengths of subfeatures. The full plDDT score color scheme displayed on the top model is shown with its expected position error plot. For this model, the ipTM = - pTM = 0.86.

bioRxiv preprint doi: https://doi.org/10.1101/2025.11.12.688109; this version posted November 12, 2025. The copyright holder for this preprint (which was not certified by peer review) is the author/funder, who has granted bioRxiv a license to display the preprint in perpetuity. It is made available under aCC-BY 4.0 International license.

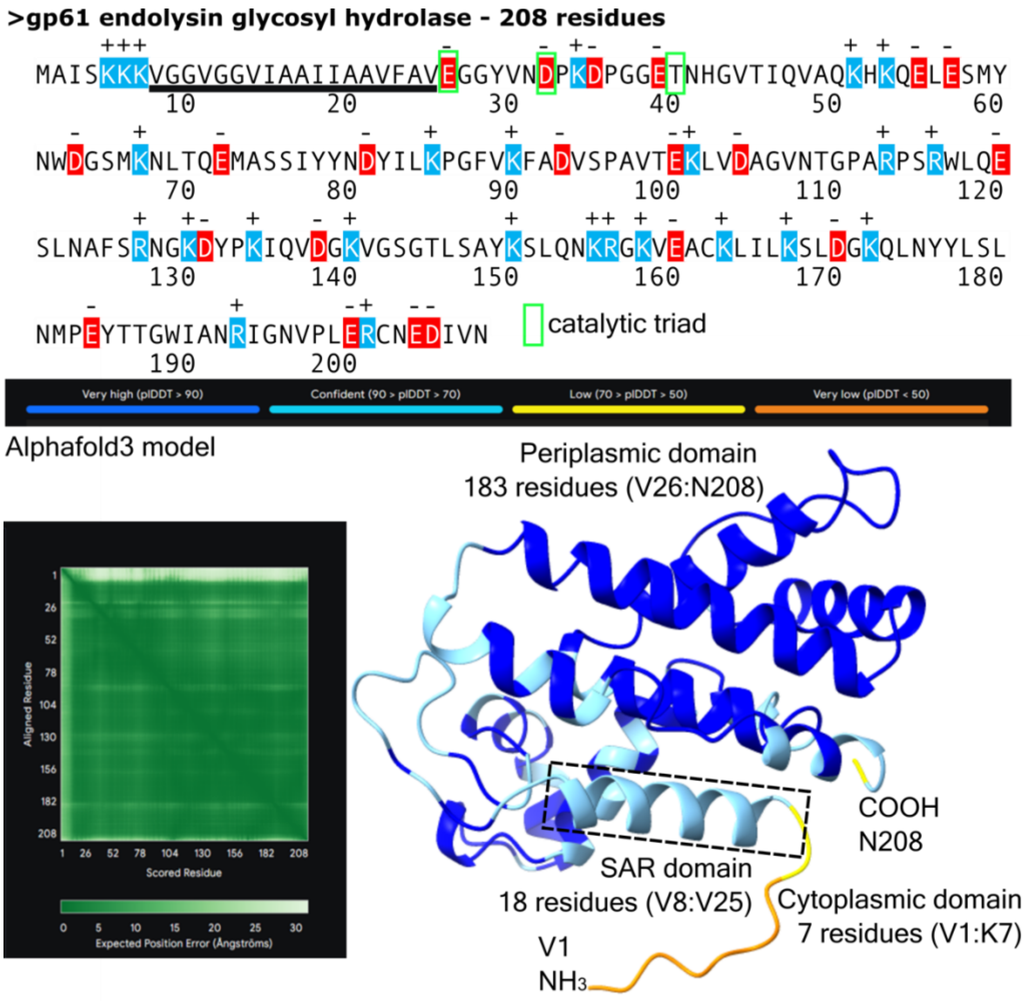

Figure S2. Predicted three-dimensional structures and topological organization of N4 spanins. The full primary sequences of gp60 and gp60a with charged residues marked, lipobox boxed, and the transmembrane domain underlined. Models for each protein were predicted using the AlphaFold3 Server (top model shown), oriented relative to the respective membrane, and annotated with lengths of subfeatures. Membrane topology was derived from TMHMM 2.0 (transmembrane helices) and SignalP 6.0 (signal peptides and cleavage sites). The full plDDT score color scheme displayed on the top models is shown with their expected position error plots. For these models, the ipTM = - pTM = 0.61 and ipTM = - pTM = 0.24 for gp60 and gp60a, respectively.

bioRxiv preprint doi: https://doi.org/10.1101/2025.11.12.688109; this version posted November 12, 2025. The copyright holder for this preprint (which was not certified by peer review) is the author/funder, who has granted bioRxiv a license to display the preprint in perpetuity. It is made available under aCC-BY 4.0 International license.

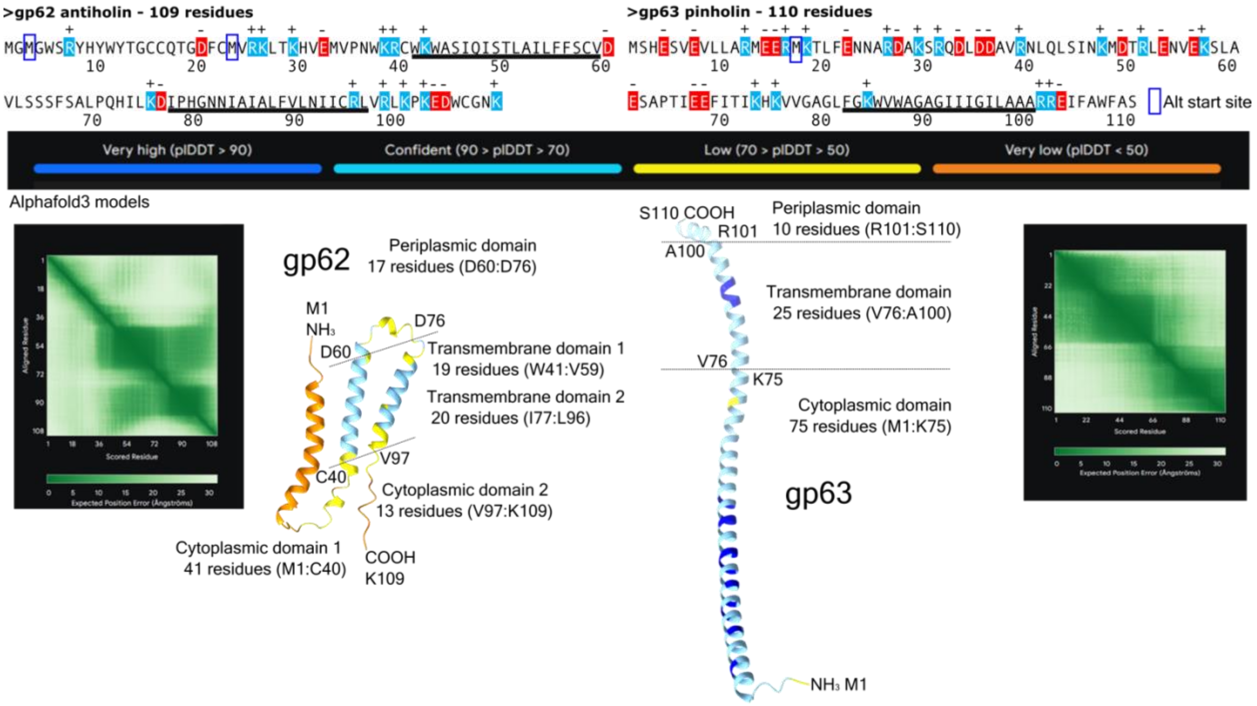

Figure S3. Predicted three-dimensional structures and topological organization of N4 holin and lysis regulator proteins. The full primary sequences of gp62 and gp63 with charged residues marked, transmembrane domains underlined, and alternative start sites boxed. Models for each protein were predicted using AlphaFold3 Server, oriented relative to the respective membrane, and annotated with subfeature lengths. Membrane topology was derived from TMHMM 2.0 (transmembrane helices). The full plDDT score color scheme displayed on the top models is shown with their expected position error plots. For these models, the ipTM = - pTM = 0.35 and ipTM = - pTM = 0.41 for gp62 and gp63, respectively.

Movies S1 and S2. N4 wildtype infection lysis videos. MG1655 was infected with N4 WT at an MOI=5 and aerated at 37°C. Infected cells were imaged at 100x magnification in time series ~180 minutes post-infection. Videos are rendered at 11 fps.

Table S1. Table describing nucleotide and coding differences between Refseq, lab stock N4 WT, and lab stock N4 r phage genome sequences. The lab N4 stocks (WT, 70,156 bp and two *r* derivatives) sequenced via Illumina technology were compared to the NCBI Refseq sequence (NC_008720.1, 70,153 bp) derived from (Genbank EF056009).

Genomic Feature	RefSeq	bp	Lab Stock N4 WT	bp	Lab Stock N4 *r*
Noncoding in Direct Terminal Repeat before gp1	9 cytosines	32-40	C <u>insertion</u>	40	CC or CCCC insertions

bioRxiv preprint doi: https://doi.org/10.1101/2025.11.12.688109; this version posted November 12, 2025. The copyright holder for this preprint (which was not certified by peer review) is the author/funder, who has granted bioRxiv a license to display the preprint in perpetuity. It is made available under aCC-BY 4.0 International license.

Gp20, predicted protease modulator	CAT, H110	8584	CGT, R110	8585	Same as lab WT stock
Gp47, unknown function	-	32048	A insertion, frameshift in K124 out of 145 aa, results in 147 aa product	32050	Same as lab WT stock
Noncoding region	A	33565	T	33567	Same as lab WT stock
Gp50	Same as lab WT stock	42668	TTG, L786	42670	TTA, L786 (silent)
Gp53, unknown function	CTG, Q98	49902	CGG, R98	49904	Same as Refseq
Gp59, portal protein	CTT, L27	56888	CCT, P27	56890	Same as lab WT stock
Noncoding hairpin between gp65 and gp64	-	60035	G insertion	60038	Same as Refseq

Table S2. Accessions of gp62 and 63 proteins hits. Genbank protein accessions for BLASTp and BLASTx hits and manually added proteins. For unannotated genes, the genome accession is given with the coordinates of the aligned ORF. Due to deduplication to remove 100% identical sequences, not all proteins in the table are shown in alignments.

**Organisms**	**gp62 matches in phage**	**gp62 matches in phage & bacteria**	**gp63 matches in phage**	**gp63 matches in phage & bacteria**
Achromobacter_phage_JWAlpha	AHC94033.1	AHC94033.1	YP_009004782.1	YP_009004782.1
Achromobacter_phage_JWDelta	AHC56593.1	AHC56593.1	AHC56594.1	AHC56594.1
Achromobacter_phage_phiAxp-3	ALA45535.1	ALA45535.1	YP_009208719.1	YP_009208719.1
Achromobacter_phage_vB_AxyP_19-32_Axy04	QDH83786	QDH83786	YP_010079358.1	YP_010079358.1
Achromobacter_phage_vB_AxyP_19-32_Axy10	YP_010079357.1	YP_010079357.1	YP_010079358.1	YP_010079358.1
Achromobacter_phage_vB_AxyP_19-32_Axy11	YP_010079439.1	YP_010079439.1	YP_010079440.1	YP_010079440.1
Achromobacter_ruhlandii		WP_083036935.1		
Achromobacter_xylosoxidans		MFY1939543.1		
Achromobacter_xylosoxidans		GAB1835574.1		
Achromobacter_xylosoxidans		WP_241123031.1		
Achromobacter_xylosoxidans		WP_241060487.1		
Achromobacter_xylosoxidans		MFY2995853.1		
Angelakisella_sp.		MEG0944652.1		MEG0944653.1
Bacteriophage_sp.	DAE39493.1	DAE39493.1	DAE39494.1	DAE39494.1

bioRxiv preprint doi: https://doi.org/10.1101/2025.11.12.688109; this version posted November 12, 2025. The copyright holder for this preprint (which was not certified by peer review) is the author/funder, who has granted bioRxiv a license to display the preprint in perpetuity. It is made available under aCC-BY 4.0 International license.

Burkholderia_multivorans		WP_217094029.1		
Burkholderia_phage_vB_BpP_HN01	UNI71511.1	UNI71511.1	UNI71512.1	UNI71512.1
Caballeronia_sp._LZ034LL				WP_309821259.1
Caballeronia_sp._TF1N1				WP_244832408.1
Candidatus_Zhuqueibacterota_bacterium				NUM72746.1
Caudoviricetes_sp.	DAH87457.1	DAH87457.1	DAH87456.1	DAH87456.1
Caudoviricetes_sp.	DAE75200.1	DAE75200.1	DAE75201.1	DAE75201.1
Caudoviricetes_sp.	QHJ80072.1	QHJ80072.1	QHJ80071.1	QHJ80071.1
Caudoviricetes_sp.	QHJ80356.1	QHJ80356.1	QHJ80357.1	QHJ80357.1
Caudoviricetes_sp.	XOR47523.1	XOR47523.1	XOR47522.1	XOR47522.1
Caudoviricetes_sp.			DAG71655.1	DAG71655.1
Caudoviricetes_sp.	QHJ78132.1	QHJ78132.1	QHJ78131.1	QHJ78131.1
Caudoviricetes_sp.	XOR20447.1	XOR20447.1	XOR20446.1	XOR20446.1
Cohaesibacteraceae_bacterium				MBL4787369.1
Delftia_phage_RG-2014			YP_009148444.2	YP_009148444.2
Dyella_japonica_DSM_16301				KLD61728.1
Enterococcus_faecium		WP_161985582.1		WP_202915382.1
Erwinia_phage_Ea9-2	AHI60142.1	AHI60142.1	YP_009007460.1	YP_009007460.1
Erwinia_phage_Fifi287	XHG84652.1	XHG84652.1	XHG84651.1	XHG84651.1
Erwinia_phage_phiEaP8	AWN06198.1	AWN06198.1	YP_009889561.1	YP_009889561.1
Erwinia_phage_vB_EamP_Frozen	ANJ65212.1	ANJ65212.1	YP_009286213.1	YP_009286213.1
Escherichia_coli				WP_241362633.1
Escherichia_coli				HCJ8179830.1
Escherichia_phage_Bp4	YP_009031977.1	YP_009031977.1	YP_009031976.1	YP_009031976.1
Escherichia_phage_EC1-UPM	YP_009598342.1	YP_009598342.1	YP_009598343.1	YP_009598343.1
Escherichia_phage_ECBP1	JX415535 60,285..60,608	JX415535 60,285..60,608		
Escherichia_phage_IME11	YP_006990609.1	YP_006990609.1	YP_006990608.1	YP_006990608.1
Escherichia_phage_KKP_3715	WLW40929.1	WLW40929.1	WLW40928.1	WLW40928.1
Escherichia_phage_Mimir124	XLZ23719.1	XLZ23719.1	XLZ23720.1	XLZ23720.1
Escherichia_phage_N4	YP_950540.1	YP_950540.1	YP_950541.1	YP_950541.1
Escherichia_phage_nithesis	XRM24118.1	XRM24118.1	XRM24117.1	XRM24117.1
Escherichia_phage_PBM-3	XQQ52605.1	XQQ52605.1	XQQ52604.1	XQQ52604.1
Escherichia_phage_PGN829.1	MH733496 33,557..33,880	MH733496 33,557..33,880	YP_010659725.1	YP_010659725.1

bioRxiv preprint doi: https://doi.org/10.1101/2025.11.12.688109; this version posted November 12, 2025. The copyright holder for this preprint (which was not certified by peer review) is the author/funder, who has granted bioRxiv a license to display the preprint in perpetuity. It is made available under aCC-BY 4.0 International license.

Escherichia_phage_phi_G17	YP_010659598.1	YP_010659598.1	YP_010659597.1	YP_010659597.1
Escherichia_phage_RudolfBernhard_Bas96	XPK42786.1	XPK42786.1	XPK42785.1	XPK42785.1
Escherichia_phage_St11Ph5	YP_010659923.1	YP_010659923.1	YP_010659924.1	YP_010659924.1
Escherichia_phage_U1G	MZ394712 61,087..61,428	MZ394712 61,087..61,428	YP_010659834.1	YP_010659834.1
Escherichia_phage_UE-M6	WWE95588.1	WWE95588.1	WWE95587.1	WWE95587.1
Escherichia_phage_UE-S5a	WVP99845.1	WVP99845.1	WVP99844.1	WVP99844.1
Escherichia_phage_UE-S5b	WWE95417.1	WWE95417.1	WWE95416.1	WWE95416.1
Escherichia_phage_vB_Ec-G1			XKC19818.1	XKC19818.1
Escherichia_phage_vB_Eco_F22	WBF79724.1	WBF79724.1	WBF79729.1	WBF79729.1
Escherichia_phage_vB_EcoM_PD205	UVK80513.1	UVK80513.1	UVK80512.1	UVK80512.1
Escherichia_phage_vB_EcoP_CU10EC	XKC24791.1	XKC24791.1	XKC24790.1	XKC24790.1
Escherichia_phage_vB_EcoP_G7C	YP_004782193.1	YP_004782193.1	YP_004782194.1	YP_004782194.1
Escherichia_phage_vB_EcoP_PhAPEC5	NC_024786 59,970..60,287	NC_024786 59,970..60,287	YP_009055576.1	YP_009055576.1
Escherichia_phage_vB_EcoP_PhAPEC7	YP_009056198.1	YP_009056198.1	YP_009056199.1	YP_009056199.1
Escherichia_phage_vB_EcoP_PW8	XLQ29994.1	XLQ29994.1	XLQ29993.1	XLQ29993.1
Escherichia_phage_vB_EcoP_ShWW44	XOL08158.1	XOL08158.1	XOL08159.1	XOL08159.1
Escherichia_phage_vB_EcoP_SP5M	NC_070869 61,411..61,734	NC_070869 61,411..61,734	YP_010659583.1	YP_010659583.1
Escherichia_phage_vB_EcoP-ZQ2	YP_010659967.1	YP_010659967.1	YP_010659966.1	YP_010659966.1
Escherichia_phage_vB_EcoS_Uz-1	UWJ04308.1	UWJ04308.1	UWJ04307.1	UWJ04307.1
Escherichia_phage_VTCCBPA_322	XFC51918.1	XFC51918.1	XFC51917.1	XFC51917.1
Hyphomicrobiales_bacterium				PCH80090.1
Klebsiella_phage_KP8	YP_009837521.1	YP_009837521.1	YP_009837522.1	YP_009837522.1
Klebsiella_phage_phi1_146050	XCO41099.1	XCO41099.1	XCO41098.1	XCO41098.1
Klebsiella_phage_vB_KoxiM_BaqKoxi	XOD32595.1	XOD32595.1	XOD32660.1	XOD32660.1
Klebsiella_phage_vB_Kpn16-P2	XCG96197.1	XCG96197.1	XCG96198.1	XCG96198.1
Klebsiella_phage_vB_KpnP_PW7	XLZ24159.1	XLZ24159.1	XLZ24160.1	XLZ24160.1
Klebsiella_phage_VLCpiP4a	UVX30947.1	UVX30947.1	UVX30946.1	UVX30946.1
Klebsiella_phage_ZCKP1			YP_009803405.1	YP_009803405.1
Martelella_endophytica				WP_045679234.1

bioRxiv preprint doi: https://doi.org/10.1101/2025.11.12.688109; this version posted November 12, 2025. The copyright holder for this preprint (which was not certified by peer review) is the author/funder, who has granted bioRxiv a license to display the preprint in perpetuity. It is made available under aCC-BY 4.0 International license.

Martelella_mangrovi				WP_354434926.1
Martelella_sp.				WP_347445848.1
Methylophaga_sp.				MAX51271.1
Nitratireductor_rhodophyticola				WP_349366794.1
Paraburkholderia_sp._SIMBA_0 54				WP_410857233.1
Podoviridae_sp._ctda_1	MH622920 43,231..43,533	MH622920 43,231..43,533	AXH72017.1	AXH72017.1
Pseudomonas_phage_Arace01	XAI69743.1	XAI69743.1	XAI69744.1	XAI69744.1
Pseudomonas_phage_inbricus	YP_01007924 2.1	YP_010079242.1	YP_0100792 43.1	YP_010079243.1
Pseudomonas_phage_Zuri	YP_00988304 2.1	YP_009883042.1	YP_0098830 43.1	YP_009883043.1
Rhizobium_phage_RHph_X2_2 8B	YP_01065848 3.1	YP_010658483.1	YP_0106584 84.1	YP_010658484.1
Roseibium_sp.				MEP2706519.1
Salmonella_enterica		WP_265323802.1		WP_265323801.1
Salmonella_enterica_subsp._ent erica_serovar_Meleagridis		EBO1428181.1		EBO1428180.1
Shigella_phage_Moonfish	XNM21675.1	XNM21675.1	XNM21674. 1	XNM21674.1
Shigella_phage_pSb-1	YP_00900841 6.1	YP_009008416.1	YP_0090084 17.1	YP_009008417.1
Shigella_virus_Moo19	YP_01065780 1.1	YP_010657801.1	YP_0106578 02.1	YP_010657802.1
Sphingobium		WP_099232467.1		
Sphingobium		WP_037510357.1		
Sphingobium_sp.		WP_422247209.1		
Sphingobium_yanoikuyae		WP_122129433.1		
Sphingomonas_paucimobilis		WP_047866766.1		
Sphingomonas_sanguinis		WP_153006113.1		
Sphingomonas_sp._Leaf4		WP_230769790.1		
Sinorhizobium_phage_ort11			YP_0098843 77.1	YP_009884377.1
Sphaerochaetaceae_bacterium				MFA7188451.1
Stenotrophomonas_phage_Paxi	YP_01065940 3.1	YP_010659403.1	YP_0106594 04.1	YP_010659404.1
Stenotrophomonas_phage_Pokk en	YP_00988370 8.1	YP_009883708.1	YP_0098837 09.1	YP_009883709.1
unclassified_Martelella				WP_180901931.1
uncultured_Martelella_sp.				WP_319519708.1
unclassified_Sphingomonas		WP_055756566.1		
uncultured_Sphingomonas_sp.		WP_298810650.1		
Vreelandella_profundi				WP_237673536.1
Wisapiscaud_virus_1	XJU74176.1	XJU74176.1	XJU74175.1	XJU74175.1

bioRxiv preprint doi: https://doi.org/10.1101/2025.11.12.688109; this version posted November 12, 2025. The copyright holder for this preprint (which was not certified by peer review) is the author/funder, who has granted bioRxiv a license to display the preprint in perpetuity. It is made available under aCC-BY 4.0 International license.

Xanthomonas_phage_RiverRider	YP_009837849.1	YP_009837849.1	YP_009837850.1	YP_009837850.1
Xanthomonas_virus_PB119	UZV39858.1	UZV39858.1	UZV39859.1	UZV39859.1
